# Nutritional intake and growth in very low birth weight infants: a comparison of standardized versus parent-led complementary feeding

**DOI:** 10.1038/s41430-026-01809-z

**Published:** 2026-08-08

**Authors:** Melanie Gsoellpointner, Margarita Thanhaeuser, Margit Kornsteiner-Krenn, Fabian Eibensteiner, Robin Ristl, Bernd Jilma, Sophia Brandstetter, Angelika Berger, Nadja Haiden

**Affiliations:** 1 Department of Neonatology, Kepler University Hospital, Johannes Kepler University, Linz, Austria; 2 Department of Pediatrics and Adolescent Medicine, Comprehensive Center for Pediatrics, Medical University of Vienna, Vienna, Austria; 3 Center for Medical Data Science, Medical University of Vienna, Vienna, Austria; 4 Department of Clinical Pharmacology, Medical University of Vienna, Vienna, Austria

**Keywords:** Randomized controlled trials, Nutrition, Paediatrics

## Abstract

The complementary feeding (CF) period is critical for ensuring adequate nutrient intake to support optimal growth in very low birth weight (VLBW) infants. This analysis compares nutritional intake and growth across two CF strategies: a standardized, protocol-driven approach and a non-standardized, parent-led approach. Both cohorts included infants with a birth weight <1500 g. The standardized group followed an age-dependent CF protocol utilizing commercial baby jars, whereas the non-standardized cohort practiced parent-led feeding without dietary restrictions. Nutritional intake was assessed using 3-day dietary records. Infants in the non-standardized study had significantly higher intakes of protein, fat, arachidonic and docosahexaenoic acid, while the standardized cohort showed higher iron and zinc intakes from 6 months corrected age onwards. Despite nutritional differences, growth was similar between cohorts. This comparison demonstrates notable differences in nutrient intake between CF approaches and may guide individualized strategies to support the nutritional needs of VLBW infants. Clinical trial registration: Randomized, standardized study: ClinicalTrials.gov: NCT01809548. Non-standardized, prospective study: ClinicalTrials.gov: NCT02936219.

## Introduction

Very low birth weight (VLBW) infants have higher nutritional needs that often persist beyond hospital discharge, making adequate nutrition during complementary feeding (CF) essential for optimal growth [1]. However, structured guidelines on CF composition for former preterm infants remain limited.

Existing trials have primarily focused on the timing of solid food introduction and were conducted in low-resource settings or prior to the availability of post-discharge formulas, limiting clinical relevance [2, 3]. More recent studies in former preterm infants have not assessed nutritional intake [4].

Our group previously examined CF timing in two cohorts using different feeding approaches: a standardized approach using commercial baby jars and a parent-led approach without dietary restriction [5, 6].

To date, no studies have compared standardized versus non-standardized CF in terms of nutrient intake and growth in preterm infants. This analysis therefore evaluates whether different CF strategies influence nutritional intake and growth, with implications for parental guidance and future recommendations for VLBW infants.

## Methods

This analysis included two studies: a randomized trial (standardized study) and a prospective observational study (non-standardized), both enrolling VLBW infants (birth weight <1500 g) followed at the outpatient clinic of the Division of Neonatology, Medical University of Vienna. Infants with major congenital anomalies were excluded in both studies. For this secondary analysis, eligibility criteria were harmonized across both datasets to ensure comparability. Accordingly, infants with necrotizing enterocolitis (NEC) grade >II and bronchopulmonary dysplasia (BPD) were additionally excluded from the observational cohort. Intraventricular hemorrhage (IVH) was not an exclusion criterion in the randomized trial and was therefore not applied as an exclusion criterion in this analysis. In the randomized study, infants received a standardized CF regimen, introduced early at 10–12 or late 16–18 weeks corrected age (CA). Age-appropriate commercial foods were provided until 12 months CA, with monthly dietary intake assessed using 3-day dietary records.

The observational study reflected non-standardized, parental practices, with infants stratified to an early or late CF introduction group (</≥17 weeks CA). Growth was measured at each visit from term-equivalent age to 12 months CA. Dietary intake was assessed with 3-day food records, including both commercial and homemade foods. Nutrient analysis in both cohorts used the same software (nut.s, Vienna, Austria; Version II.3.1) and methodology [7, 8].

Nutritional intake at 3, 6, 9, and 12 months CA and growth from birth to 12 months CA were compared. The primary outcome was protein intake (g/kg/day); secondary outcomes included intakes of energy, fatty acids (DHA, AA), micronutrients (iron, vitamin D, calcium, phosphorus, zinc, iodine, vitamin B12) and growth outcomes.

Both studies were approved by the Ethics Committee of the Medical University of Vienna (EK: 1744/2012, EK: 1273/2016) and registered on ClinicalTrials.gov (NCT01809548, NCT02936219). Detailed study design and primary outcomes of both studies have been published previously [5, 6].

Statistical analyses used linear mixed-effects models adjusted for sex, gestational age, CF timing, and feeding type at discharge. Within each study, randomized and stratified groups were combined into a single cohort. Baseline characteristics were compared using non-parametric and categorical tests. Significance was set at *p* < 0.05 with correction for multiple testing. Analyses were performed in R 4.1.1 (R Core Team, 2022).

## Results

### Study participants & baseline characteristics

Baseline characteristics are summarized in Table 1. 177 infants in the standardized study and 176 of 218 infants from the initial cohort in the non-standardized study were included.

**Table 1:** Baseline characteristics & dietary records.

Parameter	Standardized study (*n* = 177)	Non-standardized study (*n* = 176)	*p*-value
*Neonatal parameters*			
Male sex	98 (55%)	96 (54%)	0.96
Gestational age (weeks)	27 + 2 (25 + 2–28 + 5)	27 + 2 (25 + 4–29 + 0)	0.80
Gestational age (days)	191 (177–201)	191 (179–203)	0.80
Birth weight (g)	930 (725–1140)	978 (754–1170)	0.29
Birth head circumference (cm)	25 (23–26.5)	25 (23–26.5)	0.48
Birth height (cm)	35 (32–37)	35 (33–37.5)	0.50
*Neonatal morbidities*			
IVH Grade I + II	11 (6%)	18 (10%)	0.60
IVH Grade III + IV	10 (6%)	14 (8%)	
NEC grade I + II	4 (2%)	10 (6%)	0.17
*Obstetric and parental parameters*			
Multiple pregnancy	60 (34%)	55 (31%)	0.68
Cesarean delivery	162 (92%)	151 (86%)	0.13
Age of mother at birth	32 (29–37)	33 (29–36)	0.97
*Nutrition*			
Introduction of solid foods (weeks CA)	12.0 (10.0–16.0)	15.4 (13.2–18.9)	<0.001
Type of milk-feeding at discharge			
Breastfed	51 (29%)	96 (55%)	<0.001
Formula-fed	60 (34%)	46 (26%)	
Mixed-fed	63 (36%)	34 (19%)	
*Available 3-day dietary records*			
3 months CA	127 (72%)	132 (75%)	/
6 months CA	128 (72%)	125 (71%)	
9 months CA	116 (66%)	89 (51%)	
12 months CA	112 (63%)	96 (55%)	

Categorical data are presented as numbers with percentages in parentheses. Continuous data are presented as the median and interquartile range in parentheses. *CA* corrected age; p-values < 0.05 were considered statistically significant and marked bold. *IVH* Intraventricular haemorrhage, *NEC* Necrotizing enterocolitis.

Median birth weight and gestational age were comparable between the two study cohorts (930–978 g; 27 + 2 weeks). Notably, solids were introduced earlier in the standardized cohort (12.0 vs. 15.4 weeks CA, *p* < 0.001).

#### Nutritional intake

**Fig. 1:**
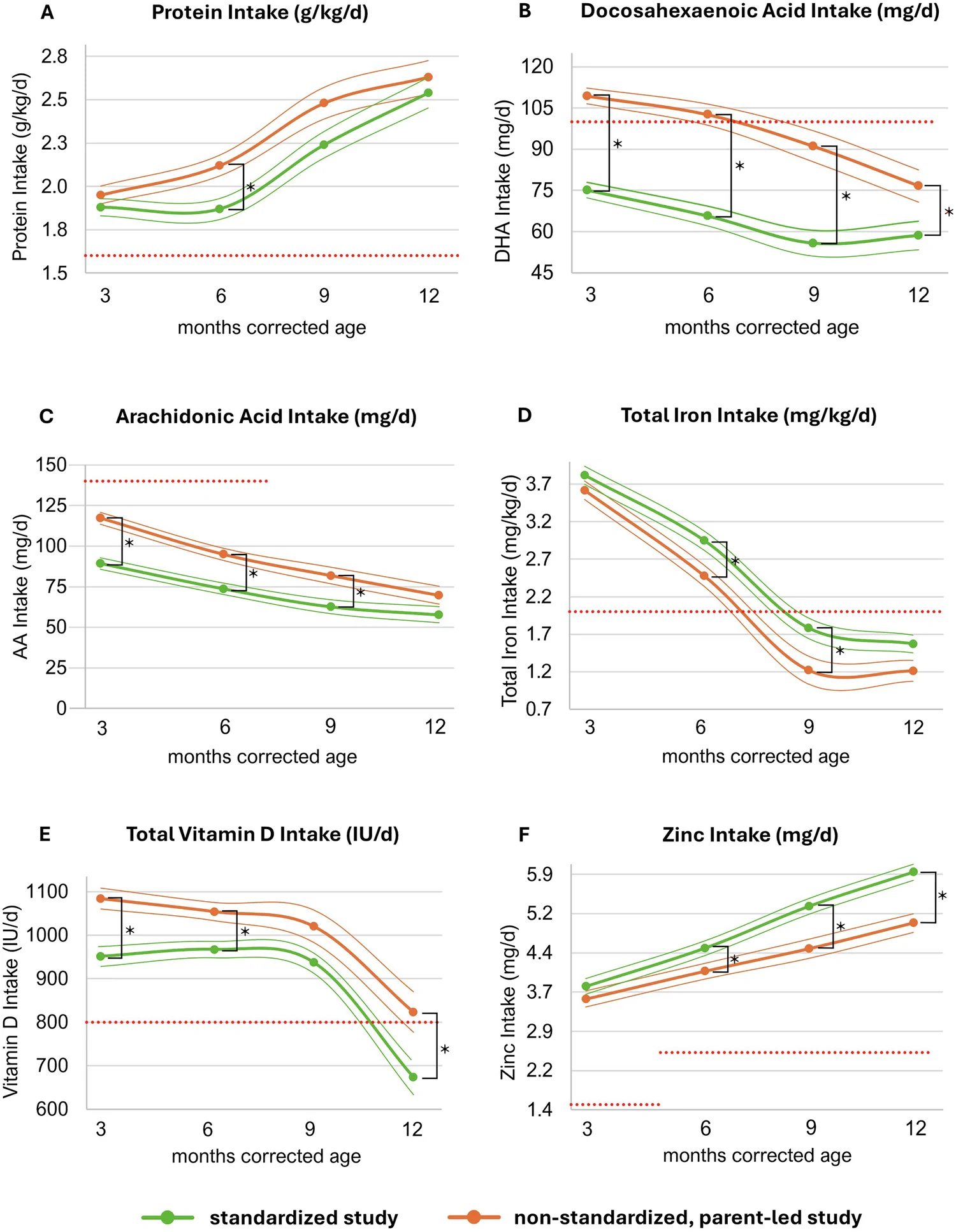
Nutritional intake comparing standardized versus non-standardized complementary feeding in VLBW infants. Nutritional intake of Protein (**A**), DHA (**B**), AA (**C**), total iron (**D**), Vitamin D (**E**) and Zinc (**F**) comparing standardized versus non-standardized complementary feeding in VLBW-infants Red lines indicate references values, green lines the specific nutrient.

At 3 months CA, mean energy intake (kcal/d) was significantly lower in the standardized study (496 ± 9 kcal/d) compared to the non-standardized study (542 ± 9 kcal/d; *p*-adj. < 0.001), but values aligned thereafter. Protein intake (g/kg/d) was higher in the non-standardized study across all timepoints compared to the standardized study, significantly at 6 months CA (standardized: 1.87 ± 0.06 g/kg/d; non-standardized: 2.12 ± 0.06 g/kg/d; *p*-adj. = 0.02) (Fig. 1A). Fat intake (g/d) was significantly higher in the non-standardized study at all observed timepoints, ranging between 25.5–27.1 g/d in the standardized study and 28.9–31.6 g/d in the non-standardized study. Carbohydrate intake (g/d) was significantly higher in the standardized study at 3 months CA (55.6 ± 1.0 g/kg/d vs. 60.0 ± 1.1 g/kg/d; *p*-adj. = 0.006), but lower at 12 months CA in the non-standardized study (121.0 ± 3.0 g/kg/d vs. 105.0 ± 3.4 g/kg/d; *p*-adj. = 0.002).

AA and DHA intakes were both higher in the non-standardized cohort (AA: 3–9 months CA (81.8–117.2 mg/d vs. 62.6–89.3 mg/d; DHA: 3-12 months CA (76.6–109.4 mg/d vs. 55.8–75.1 mg/d); Fig 1B, C). AA remained below recommended levels (140 mg/d) at all timepoints, while DHA met recommendations (100 mg/d) at 3 and 6 months CA in the non-standardized study but were inadequate in both cohorts at later stages [9, 10].

Furthermore, total vitamin D, phosphorus and vitamin B12 were higher in the non-standardized study, while total iron and zinc were significantly higher in the standardized cohort (9 months: 5.29 ± 0.15 vs. 4.48 ± 0.18 mg/d; Fig. 1D–F).

#### Growth

Despite differences in nutritional intake, anthropometric outcomes were largely similar between the two studies. Length at term-equivalent age was numerically higher in the non-standardized cohort, but this difference was not significant after adjustment (*p*-adj. = 0.19).

Complete data on nutrient intake and growth trajectories are available in the Supplementary Material.

## Discussion

This analysis demonstrates that the CF approach influences nutrient intake but not growth in VLBW infants. Infants following a parent-led, non-standardized diet showed higher intakes of protein, fat, DHA, AA, total vitamin D, phosphorus, and vitamin B12, likely reflecting the greater diversity and nutrient density of homemade and fortified foods. Conversely, standardized feeding provided higher intakes of iron and zinc from 6 months CA onwards, highlighting the benefits of structured commercial foods for critical micronutrients. Despite these differences, anthropometric outcomes were similar at 12 months CA, suggesting that both approaches can support adequate growth. Notably, DHA and AA remained below recommended levels in both groups, emphasizing the need to increase long-chain polyunsaturated fatty acid intake throughout the CF period. Overall, this comparison demonstrates notable differences in nutrient intake between CF approaches and may inform individualized nutritional strategies in VLBW infants.

However, the higher proportion of breastfed infants and later initiation of CF in the non-standardized cohort should be considered, as both may have influenced early nutrient exposure and intake patterns. In addition, this analysis is based on independent cohorts rather than a prospective head-to-head trial, and both cohorts included infants with severe IVH, a condition that may affect growth and nutrient intake. This should be considered when interpreting the findings. Future prospective studies are needed to directly compare CF approaches and optimize nutritional strategies in this population.

## Supplementary information

Supplemental Material

## Data Availability

The dataset generated and/or analyzed during the current study is available from the corresponding author upon request.
