# Supplementary material for: Nutritional intake and growth in very low birth weight infants: a comparison of standardized versus parent-led complementary feeding
Source: Eur J Clin Nutr. 2026 Aug 8;80(10):1105–8. doi: 10.1038/s41430-026-01809-z (PMC13690594; doi:10.1038/s41430-026-01809-z)
Supplement: Supplementary file 1 [file 41430_2026_1809_MOESM1_ESM.pdf]

Suppl. table 1: Macronutrient Intakes comparing the standardized and the non-standardized study.

CA	Energy (kcal/d)			Protein (g/kg/d)		
	Standardized study	Non-standardized study	p-adj.	Standardized study	Non-standardized study	p-adj.
	Mean ± SE			Mean ± SE		
3 months	496 (±9)	542 (±9)	<0.001	1.88 (±0.05)	1.95 (±0.05)	0.41
6 months	606 (±12)	642 (±12)	0.07	1.87 (±0.06)	2.12 (±0.06)	0.02
9 months	720 (±16)	746 (±18)	0.41	2.24 (±0.08)	2.48 (±0.09)	0.08
12 months	823 (±19)	802 (±21)	0.48	2.54 (±0.09)	2.63 (±0.10)	0.48

CA	Protein (g/d)			Fat (g/d)		
	Standardized study	Non-standardized study	p-adj.	Standardized study	Non-standardized study	p-adj.
	Mean ± SE			Mean ± SE		
3 months	9.8 (±0.2)	10.9 (±0.2)	0.003	26.2 (±0.5)	28.9 (±0.5)	<0.001
6 months	12.9 (±0.4)	15.0 (±0.5)	0.003	25.5 (±0.6)	29.1 (±0.6)	<0.001
9 months	17.6 (±0.6)	20.4 (±0.7)	0.003	26.0 (±1.0)	30.2 (±1.2)	0.009
12 months	22.8 (±0.8)	23.7 (±0.9)	0.46	27.1 (±0.8)	31.6 (±0.9)	<0.001

CA	Carbohydrates (g/d)			AA (mg/d)		
	Standardized study	Non-standardized study	p-adj.	Standardized study	Non-standardized study	p-adj.
	Mean ± SE			Mean ± SE		
3 months	55.6 (±1.0)	60.0 (±1.1)	0.006	89.3 (±3.5)	117.2 (±3.6)	<0.001
6 months	81.5 (±1.8)	80.7 (±1.9)	0.74	73.7 (±3.5)	94.9 (±3.7)	<0.001
9 months	104.4 (±2.7)	98.7 (±3.2)	0.23	62.6 (±4.3)	81.8 (±5.1)	0.006
12 months	121.0 (±3.0)	105.0 (±3.4)	0.002	57.7 (±4.9)	69.7 (±5.5)	0.11

CA	DHA (mg/d)		
	Standardized study	Non-standardized study	p-adj.
	Mean ± SE		
3 months	75.1 (±2.8)	109.4 (±2.9)	<0.001
6 months	65.7 (±3.6)	102.6 (±3.9)	<0.001
9 months	55.8 (±4.7)	91.1 (±5.7)	<0.001
12 months	58.6 (±5.2)	76.6 (±5.8)	0.02

SE: Standard error; p-adj: adjusted p-value (Bonferroni-Holm).

Suppl. table 2: Micronutrient Intakes comparing the standardized and the non-standardized study.

CA	Iron (mg/kg/d)			Iron total (mg/kg/d)		
	Standardized study	Non-standardized study	p-adj.	Standardized study	Non-standardized study	p-adj.
	Mean ± SE			Mean ± SE		
3 months	0.74 (±0.04)	0.68 (±0.04)	0.21	3.82 (±0.12)	3.62 (±0.12)	0.25
6 months	0.80 (±0.03)	0.68 (±0.03)	0.004	2.95 (±0.13)	2.48 (±0.14)	0.03
9 months	0.92 (±0.03)	0.68 (±0.04)	<0.001	1.78 (±0.14)	1.22 (±0.19)	0.03
12 months	0.95 (±0.03)	0.73 (±0.04)	<0.001	1.57 (±0.12)	1.21 (±0.14)	0.06

CA	Iron (mg/d)			Vitamin D (IU/d)		
	Standardized study	Non-standardized study	p-adj.	Standardized study	Non-standardized study	p-adj.
	Mean ± SE			Mean ± SE		
3 months	3.85 (±0.19)	3.82 (±0.20)	0.88	286 (±17)	335 (±18)	0.19
6 months	5.51 (±0.19)	4.79 (±0.21)	0.01	304 (±15)	297 (±16)	0.74
9 months	7.26 (±0.26)	5.60 (±0.32)	<0.001	311 (±16)	286 (±19)	0.41
12 months	8.46 (±0.30)	6.56 (±0.34)	<0.001	297 (±15)	270 (±17)	0.41

CA	Vitamin D total (IU/d)			Calcium (mg/d)		
	Standardized study	Non-standardized study	p-adj.	Standardized study	Non-standardized study	p-adj.
	Mean ± SE			Mean ± SE		
3 months	951 (±23)	1082 (±23)	<0.001	357 (±12)	402 (±12)	0.02
6 months	967 (±29)	1050 (±21)	0.006	408 (±13)	430 (±14)	0.30
9 months	937 (±25)	1022 (±36)	0.06	499 (±17)	492 (±21)	0.79
12 months	675 (±40)	829 (±45)	0.02	637 (±20)	551 (±22)	0.02

	Phosphorus (mg/d)			Zinc (mg/d)		
	Standardized study	Non-standardized study	p-adj.	Standardized study	Non-standardized study	p-adj.
	Mean ± SE			Mean ± SE		
3 months	205 (±8)	249 (±9)	<0.001	3.77 (±0.15)	3.52 (±0.15)	0.31
6 months	270 (±10)	339 (±11)	<0.001	4.49 (±0.14)	4.05 (±0.15)	0.06
9 months	361 (±12)	452 (±15)	<0.001	5.29 (±0.15)	4.48 (±0.18)	0.002
12 months	492 (±16)	531 (±18)	0.11	5.94 (±0.16)	4.97 (±0.18)	<0.001

	Iodine (µg/d)			Vitamin B12 (µg/d)		
	Standardized study	Non-standardized study	p-adj.	Standardized study	Non-standardized study	p-adj.

	Mean ± SE			Mean ± SE		
**3 months**	82.2 (±2.9)	91.5 (±3.0)	0.10	1.87 (±0.10)	1.98 (±0.10)	0.40
**6 months**	83.0 (±2.8)	86.0 (±3.0)	0.82	1.57 (±0.09)	2.02 (±0.10)	0.001
**9 months**	84.1 (±3.1)	83.3 (±3.6)	0.88	1.44 (±0.06)	1.89 (±0.08)	<0.001
**12 months**	88.1 (±3.2)	85.5 (±3.6)	0.82	1.66 (±0.07)	1.78 (±0.08)	0.37

SE: Standard error; p-adj: adjusted p-value (Bonferroni-Holm). Total iron and vitamin D reflect combined dietary and supplemental sources.

Suppl. table 3: Growth trajectories comparing the standardized and the non-standardized study.

Parameter	Timepoint	Standardized study	Non-standardized study	p-adj.
		Mean ± SE	Mean ± SE	
**Weight (g)**	birth	917 (±12)	937 (±13)	0.64
	term	3251 (±38)	3275 (±42)	0.80
	3 months CA	5752 (±68)	5634 (±75)	0.64
	6 months CA	7239 (±83)	7154 (±90)	0.80
	12 months CA	9219 (±112)	9176 (±121)	0.80
**Weight z-score**	birth	-0.10 (±0.06)	0.03 (±0.07)	0.32
	term	-0.65 (±0.07)	-0.79 (±0.08)	0.32
	3 months CA	-0.66 (±0.09)	-0.82 (±0.10)	0.32
	6 months CA	-0.51 (±0.10)	-0.69 (±0.11)	0.32
	12 months CA	-0.23 (±0.10)	-0.32 (±0.11)	0.58
**Height (cm)**	birth	34.6 (±0.2)	34.9 (±0.2)	0.45
	term	49.0 (±0.2)	49.6 (±0.2)	0.19
	3 months CA	60.1 (±0.2)	59.8 (±0.2)	0.47
	6 months CA	66.5 (±0.2)	66.8 (±0.2)	0.47
	12 months CA	74.4 (±0.2)	74.8 (±0.2)	0.45
**Height z-score**	birth	0.00 (±0.08)	0.15 (±0.08)	0.72
	term	-0.90 (±0.09)	-0.79 (±0.10)	0.72
	3 months CA	-0.40 (±0.10)	-0.49 (±0.11)	0.72
	6 months CA	-0.08 (±0.10)	-0.08 (±0.11)	0.98
	12 months CA	-0.31 (±0.09)	-0.23 (±0.10)	0.72
**Head circumference (cm)**	birth	24.5 (±0.1)	24.7 (±0.1)	0.76
	term	34.7 (±0.1)	34.7 (±0.1)	0.91
	3 months CA	39.9 (±0.1)	39.7 (±0.1)	0.76
	6 months CA	42.5 (±0.1)	42.6 (±0.1)	0.76
	12 months CA	45.3 (±0.1)	45.4 (±0.2)	0.76
**Head circumference z-score**	birth	0.14 (±0.07)	0.22 (±0.08)	0.93
	term	-0.21 (±0.08)	-0.36 (±0.09)	0.93
	3 months CA	-0.24 (±0.10)	-0.32 (±0.11)	0.93
	6 months CA	-0.21 (±0.10)	-0.22 (±0.11)	0.93
	12 months CA	-0.18 (±0.11)	-0.13 (±0.12)	0.93
